# A Single Nucleotide Mixture Enhances the Antitumor Activity of Molecular-Targeted Drugs Against Hepatocellular Carcinoma

**DOI:** 10.3389/fphar.2022.951831

**Published:** 2022-06-27

**Authors:** Da Mao, Meihong Xu, Qiyu Jiang, Huiwei Sun, Fang Sun, Ruichuang Yang, Yantao Chai, Xiaojuan Li, Boan Li, Yong Li

**Affiliations:** ^1^ Department of Nutrition and Food Hygiene, School of Public Health, Peking University, Beijing, China; ^2^ Division of Chemical Metrology and Analytical Science, National Institute of Metrology, Beijing, China; ^3^ Department of Infectious Disease, Institute of Infectious Disease, The Fifth Medical Center of Chinese PLA General Hospital, Beijing, China; ^4^ Department of Clinical Laboratory, The Fifth Medical Center of Chinese PLA General Hospital, Beijing, China

**Keywords:** hepatocellular carcinoma, single nucleotide mixture, molecular-targeted therapy, tyrosine-kinase inhibitor, immune-checkpoint inhibitor, nutritional and supportive treatment

## Abstract

New strategies for molecular-targeted drug therapy for advanced hepatocellular carcinoma (HCC) ignore the contribution of the nutritional status of patients and nutritional support to improve physical status and immunity. We aimed to elucidate the role of a single nucleotide mixture (SNM) in the anti-tumor therapy of HCC, and to explore the importance of a SNM as adjuvant therapy for HCC. Compared with a lipid emulsion (commonly used nutritional supplement for HCC patients), the SNM could not induce metabolic abnormalities in HCC cells (Warburg effect), and did not affect expression of metabolic abnormality-related factors in HCC cells. The SNM could also attenuate the lymphocyte injury induced by antitumor drugs *in vitro* and *in vivo*, and promote the recruitment and survival of lymphocytes in HCC tissues. Using HCC models in SCID (server combined immune-deficiency) mice or BalB/c mice, the SNM had anti-tumor activity, and could significantly upregulate the antitumor activity of molecular-targeted drugs (tyrosine-kinase inhibitors [TKI] and immune-checkpoint inhibitors [ICI]) against HCC. We employed research models *in vivo* and *in vitro* to reveal the anti-tumor activity of the SNM on HCC. Our findings expand understanding of the SNM and contribute to HCC (especially nutritional support) therapy.

## 1 Introduction

The main risk factor for hepatocellular carcinoma (HCC) is infection by the hepatitis-B virus (HBV) and HCV (Wang et al., 2014; Forner et al., 2018; Powell et al., 2021). China has intensified efforts to inoculate its population using anti-HBV vaccines since the 1980s (Zhang et al., 2017a; Choi et al., 2021; Wang et al., 2022) and progress has been made for symptomatic treatment of HBV-infected people. Nevertheless, there are two main challenges in HCC treatment. First, with aging populations, changing dietary habits, and metabolism-based diseases (e.g., alcoholic/non-alcoholic fatty liver disease), diabetes mellitus and obesity have become new risk factors for the occurrence and progression of HCC (Anstee et al., 2019; Younossi et al., 2019; Fujii et al., 2020; Huang et al., 2021; Llovet et al., 2021). Second, >80 million people worldwide are infected with the HBV and other hepatitis viruses in China or suffering from various chronic liver diseases related to hepatitis viruses (Chen et al., 2016; Polaris Observatory Collaborators, 2018; McGlynn et al., 2021; Sung et al., 2021; Polaris Observatory HCV Collaborators, 2022). Despite receiving symptomatic treatment for HBV infection, some patients suffer disease progression and develop HCC eventually (Chen et al., 2016; Polaris Observatory Collaborators, 2018; McGlynn et al., 2021; Sung et al., 2021; Polaris Observatory HCV Collaborators, 2022).

Most patients with HCC are diagnosed at an advanced stage, so radical treatment (e.g., liver transplantation or surgery) is not possible (Javan et al., 2020; Wang and Li, 2021; Wu et al., 2021). In general, HCC is insensitive to various cytotoxic chemotherapeutics, which further limits the treatment strategies for HCC (Kim et al., 2017). Drug-based treatment strategies for HCC are molecular-targeted drugs (MTDs), including tyrosine kinase inhibitors (TKIs) such as by sorafenib (Roskoski, 2019; Roskoski, 2020; Roskoski, 2021; Roskoski, 2022) and immune-checkpoint inhibitors (ICIs) acting on programmed cell death-1/programmed cell death-ligand 1 (PD-1/PD-L1) (Donisi et al., 2020; Giraud et al., 2021). Global multicenter, randomized, controlled clinical trials have suggested that these drugs can prolong patient survival to varying degrees, but two main problems remain. First, only a proportion of patients are sensitive to TKIs or ICIs, and these patients may develop drug resistance as treatment progresses (Zhu et al., 2017; Zongyi and Xiaowu, 2020; He et al., 2021a; Llovet et al., 2022). Second, TKIs or ICIs can cause serious toxicity and side-effects (Zhu et al., 2017; Zongyi and Xiaowu, 2020; He et al., 2021a; Llovet et al., 2022). Therefore, finding a way to achieve safe and efficacious molecular-targeted therapy for HCC is an urgent and difficult problem to be solved. Combined use of antitumor drugs with different targets (e.g., ICIs combined with TKIs) or combined use of MTDs and interventional therapy are the main treatment strategies for HCC (Feng et al., 2018a; Jia et al., 2021a; Yang et al., 2021a; Zhang et al., 2022). This treatment strategy may help to achieve more potent antitumor activity (Jia et al., 2016; Xie et al., 2017; Xie et al., 2018; Li et al., 2021), but it ignores the influence of nutritional status and the immune status of the patient upon the treatment effect.

Nucleotides are important low-molecular-weight compounds that participate in the regulation and metabolism of various substances as free nucleotides or their derivatives (Wang et al., 2021a; Guan et al., 2021; Lu et al., 2021; Santos Ferreira et al., 2021). Free mononucleotides are the main high-energy compounds involved in energy metabolism, important messengers in cellular signal transduction, and metabolic regulators of various nutrients in the body (Wang et al., 2021a; Guan et al., 2021; Lu et al., 2021; Santos Ferreira et al., 2021). Addition of exogenous nucleotides can nourish lymphocytes, promote their differentiation, and inhibit DNA damage. These actions can improve cellular immune function, humoral immune function, and monocyte–macrophage phagocytosis (Xu et al., 2013). Nucleotide supplementation in the diet can improve intestinal dysfunction induced by various factors (Cai et al., 2016a). In health, addition of exogenous nucleotides helps to maintain the structure, improve the metabolism and synthesis functions, and promote antioxidant capacity in the liver. Studies (Cai et al., 2016b) have shown that supplementation with exogenous nucleotides can alleviate hepatic oxidative stress, inhibit inflammatory responses, reverse (at least in part) the host metabolic disorder caused by excessive intake of alcohol, and change some mechanisms related to metabolism (bile acids, lipids, amino acids), thereby attenuating alcohol-induced metabolic disturbances and liver damage (Cai et al., 2016b). Therefore, exploring the potential application of mononucleotide mixtures in HCC treatment is important.

We wished to explore the antitumor activity of a mononucleotide mixture in HCC. A series of *in vitro* and *in vivo* HCC models were established. Simultaneously, lymphocytes were isolated from the peripheral blood of healthy volunteers (HVs). We found that a mixture of single nucleotides could alleviate the damage wrought by anti-tumor drugs to peripheral-blood lymphocytes (PBLs), and enhance the antitumor actions of TKIs or ICIs.

## 2 Materials and Methods

### 2.1 Cell Lines

The cell lines we used were the human HCC line MHCC97-H and mouse liver cancer cell line H22, which were purchased from National Infrastructure of Cell Resources within the Chinese Academy of Medical Sciences, and China Union Medical College, respectively, both of which are in Beijing, China. PBLs (peripheral blood lymphocytes) were obtained from healthy volunteers (HVs)/healthy blood donors. Twenty samples of peripheral blood were obtained and 2–5 × 10^9^ cells isolated per 100 ml of peripheral blood. PBLs were separated and sorted by flow cytometry to obtain T cells, B cells, and NK cells. Cluster of differentiation (CD)45^+^ components were separated. Then, CD45^+^/CD56^+^ components were separated as NK cells. CD45^+^/CD3^−^/CD19^+^ components were B cells. CD45^+^/CD3^+^/CD19^−^ components were T cells. All three cell types were frozen at −80°C. Experiments using these three types of cells were carried out separately.

### 2.2 Antitumor Drugs

The apoptosis inducer carbonyl cyanide m-chlorophenyl hydrazine (CCCP; catalog number, S6494) was obtained Selleck Chemicals (Houston, TX, United States). Some MTDs used in HCC treatment can induce damage to immune cells, including sorafenib, lenvatinib, regorafenib, and cabozantinib, and BMS-1166 is a small-molecule inhibitor of PD-L1. These drugs were synthesized by Dr. Cao Shuang of Wuhan Engineering University (Wuhan, China) and their purity was >99% according to high-performance liquid chromatography (Table 1). Some cytotoxic chemotherapy drugs can cause damage to immune cells, such as paclitaxel (S1150), doxorubicin (S1208), and etoposide (S1225). These agents were purchased from Selleck Chemicals.

**TABLE 1:** the purity of drugs in the presence work.

Drugs	Purity (%)
sorafenib	99.5
Lenvatinib	99.2
Regorafenib	99.3
cabozantinib	99.6
BMS-1166	99.2

### 2.3 Single Nucleotide Mixture

The single nucleotide mixture used in this study was 5′AMP: 5′CMP: 5′GMPNa_2_: 5′UMPNa_2_ at a ratio of 22.8:26.6:20.4:30.220.4:30.2. A nutritional supplement used in the clinic served as a control: structured lipid emulsion (C6–24) injection. The structured lipid emulsion was a gift from Prof. and Dr. Xudong Gao in the Fifth Medical Center of Chinese PLA General Hospital. The single-nucleotide mixtures were conserved in our lab and described in our previous publications (Cai et al., 2016a; Cai et al., 2016b).

### 2.4 Real-Time Reverse Transcription-Quantitative Polymerase Chain Reaction

HCC cells, tissue samples, or lymphocytes were collected. Samples were disrupted and homogenized in a tissue grinder using a tissue-lysing solution (Buffer RLT Plus; Qiagen, Stanford, VA, United States) and steel beads by vortex-shaking. The column was centrifuged (12,000 rpm, 1 min, 4°C) to remove DNA. RNA in tissues or cells was collected by sedimentation with 70% ethanol (*v*/*v*) by using the RLT RNeasy centrifugal adsorption column, RNase-free water [DEPC-treated deionized water] for elution and dissolution). RNA was reverse-transcribed into complementary-DNA using SuperScript™ IV VILO™ Master Mix (2 μl) for 10 ng of RNA in a total volume <8 μl. The PCR conditions were 25°C for 10 min, 50°C for 10 min, 85°C for 5 min, and 10°C for 10 min. Next, a one-step qPCR experiment was undertaken on a RT-PCR instrument (7500 series; Applied Biosystems; Foster City, CA, United States). Power SYBR^®^ Green RT-PCR Mix (Thermo Fisher, Waltham, MA, United States) was used in a 2× buffer system and volume of 10.0 μl. The volume of the upstream primer was 0.2 μl. The volume of the downstream primer was 0.2 μl. The volume of RT Enzyme Mix (125× buffer system) was 0.16 μl. The RNA sample in HCC cells or tumor tissues were used as the amplification template, and the volume was ∼0.2 μl, thereby making a total volume of 20.0 μl. The reverse-transcription reaction proceeded at 48°C for 30 min. The heat-activation step was done using AmpliTaq Gold^®^ DNA Polymerase (Thermo Fisher) at ∼95°C for 10 min, followed by 40 cycles of the following program: denaturation at 95°C for 15 s; annealing/extension at 60°C for 1 min; denaturation at 95°C for 15 s; extension at 60°C for 15 s. The primers used in the RT-qPCR experiments are shown in Table 2.

**TABLE 2:** Primers used in this study.

Gene	Forward	Reverse
BCL2	5′-GAT​CGT​TGC​CTT​ATG​CA TTTGTTTTG-3′	5′-CGG​ATC​TTT​ATT​TCA​TGA​GGC​ACG​TTA-3′
Survivin	5′-ACA​TGC​AGC​TCG​AAT​GAG​AA CAT-3′	5′-GAT​TCC​CAA​CAC​CTC​AAG​CCA-3′
cIAP-1	5′-GTG​TTC​TAG​TTA​ATC​CTG​AGC AGCTT-3′	5′ -TGG​AAA​CCA​CTT​GGC​ATG​TTG​A-3′
cIAP-2	5′-CAA​GGA​CCA​CCG​CAT​CT CT-3′	5′-AGC​TCC​TTG​AAG​CAG​AAG​AAA​CA-3′
E-cadherin	5′-CTC​CTG​AAA​AGA​GAG​TG GAAGTGT-3′	5′-CCG​GAT​TAA​TCT​CCA​GCC​AGT​T-3′
N- cadherin	5′-CCTGGATCGCGA GCAGATA-3′	5′-CCA​TTC​CAA​AAC​CTG​GTG​TAA​GAA​C-3′
Vimentin	5′-ACCGCACACAG CAAGGCGAT-3′	5′-CGA​TTG​AGG​GCT​CCT​AGC​GGT​T-3′
GLUT1	5′-TTGCAGGCTTCTC CAACTGGAC-3′	5′ -CAG​AAC​CAG​GAG​CAC​AGT​GAA​G-3′
HIF-1α	5′-TATGAGCCAGAAGAA CTTTTAGGC- 3′	5′-CAC​CTC​TTT​TGG​CAA​GCA​TCC​TG-3′
EPAS-1	5′-CTGTGTCTGAGAA GAGTAACTTCC-3′	5′-TTG​CCA​TAG​GCT​GAG​GAC​TCC​T-3′
Snail	5′-TGCCCTCAAGATGC ACATCCGA-3′	5′-GGG​ACA​GGA​GAA​GGG​CTT​CTC-3′
Twist	5′-GCCAGGTACATC GACTTCCTCT-3′	5′-TCC​ATC​CTC​CAG​ACC​GAG​AAG​G-3′
β-actin	5′-CAC​CAT​TGG​CAA​TGA​GCG​GTT​C-3′	5′-AGGTCTTTGCGGA GTCCACGT-3′

Relative expression of a target gene was based on the ratio of its cycle-threshold value in the RT-qPCR experiment to the cycle-threshold value of the internal reference. Then, heatmaps were drawn according to the relative expression of each target gene. In each experiment, the control group of each target gene was taken as a unit of 1, and the difference between the experimental group and control group was calculated. An increase in expression of the target gene denoted a positive fold change, and a decrease in the expression of the target gene denoted a negative fold change, and a heatmap was drawn at this point (Ma et al., 2020; Zhou et al., 2021).

### 2.5 The 3-(4,5-Dimethylthiazol-2-yl)-2,5-Diphenyltetrazolium Bromide (MTT)/Cellular Survival Assays

Cells were prepared as suspensions and seeded in 96-well culture plates. Immunotoxic drugs (sorafenib, regorafenib, lenvatinib, cabozantinib, paclitaxel, etoposide, CCCP, adriamycin) were used alone or in combination with the SNM. A solution of these drugs was prepared as described previously (Feng et al., 2020; Jia et al., 2021b; Wang et al., 2021b; Du et al., 2021). After 48 h of drug action (alone or in combination), MTT reagent was added directly to the 96-well plate, after which cells were incubated at 37°C for 5 h. The plate was centrifuged to separate cells from the liquid, and aggregated at the bottom of the plate. After discarding the supernatant, the cell sample was lysed with dimethyl sulfoxide and agitated for 5 min. Additional centrifugation was done to remove insoluble tiny debris and foam, and a Stripette™ (Corning, Corning, NY, United States) was employed to transfer the supernatant to a new 96-well plate. A multifunctional, full-wavelength microplate reader was employed to measure the absorbance of samples in each well at 490 nm. The absorbance value denoted the relative number of cells (number of viable cells/total number of cells) and reflected the damaging effect of a drug on cells (Li et al., 2017a; Guan et al., 2017; Li et al., 2018a). For experiments on cell survival, the percent inhibition caused by a drug was calculated according to the absorbance value at 490 nm using the formula (Li et al., 2017a; Guan et al., 2017; Li et al., 2018a):

$${\text{Percent inhibition }=\text{ Absorbance}}_{\text{control}}-{\text{absorbance}}_{\text{drug}}/{\text{absorbance}}_{\text{control}}\times 100\text{\%}$$

### 2.6 Apoptosis Assays

Cells were prepared as suspensions and seeded in six-well culture plates. Immunotoxic drugs (sorafenib, regorafenib, lenvatinib, cabozantinib, paclitaxel, etoposide, CCCP, adriamycin) were used alone or in combination with the SNM. After 48 h of drug action (alone or in combination), cells were aspirated from the six-well plate and added to a 6-ml centrifuge tube. Phosphate-buffered saline (PBS) was added to each centrifuge tube to make up the volume, followed by centrifugation (800 rpm for 3 min), and this step was repeated. Then, 1 ml of 1× binding buffer (cells per six-well plate were resuspended in 1 ml) was added so that the cell density was 1.0 × 10^6^/ml. Next, 2 μl of fluorescently labeled annexin V and 7-AAD (7-Aminoactinomycin D) were added to 100 μl of cells for further experiments. The cell suspension prepared in the previous step was mixed gently and allowed to incubate at room temperature for 15–20 min in the dark. After incubation, the cell sample was transferred to a flow tube and 400 µl of loading buffer added. Fluorescently labeled annexin V-positive, 7-AAD-negative cells were regarded to have undergone “early” apoptosis. Fluorescently labeled annexin V-positive, 7-AAD-positive cells were regarded as having undergone “late” apoptosis. Fluorescently labeled annexin V-negative, 7-AAD-positive cells were regarding as having undergone “necrosis” (Ma et al., 2015). Total apoptosis of cells was calculated as (Ma et al., 2015):

$${\text{Total apoptosis }=\text{ Cells}}_{\text{early apoptosis}}{\;+\text{ Cells}}_{\text{late apoptosis}}$$

Heatmaps were drawn according to percent inhibition of drugs and total apoptosis (Ma et al., 2020; Zhou et al., 2021).

### 2.7 Animal Experiments

#### 2.7.1 Experimental Animals and Agents

The study protocol was approved by the Animal Ethics Committee of the Fifth Medical Center of the Chinese People’s Liberation Army General Hospital (Beijing, China). We used immunodeficient mice (SCID mice: deletion of T cells and B cells) and normal Bal B/c mice, both of which were from Beijing Speifu Biotechnology (Beijing, China). Mice were fed American Institute of Nutrition-1993 Maintenance (AIN-93M) chow. Mice underwent inhalation anesthesia using isoflurane (Shenzhen Wo Ruide, Shenzhen, China). *In vivo* imaging of mice was done in the Department of Nuclear Medicine within Peking University Cancer Hospital (Beijing, China).

#### 2.7.2 Oral Formulations of TKIs or ICIs (Liu et al., 2021a; Yang et al., 2021b)

A precision (1/10,000 precision) balance was used to weigh 20–50 mg of pure powders of sorafenib or BMS-1166. Sorafenib or BMS-1166 were mixed with polyethylene glycol 400, Tween 80, and a small volume of dimethyl sulfoxide supplemented by ultrasound and vortex-mixing. After dissolution, sterilized physiological (0.9%) saline was added, followed by stirring and shaking to obtain an oral preparation of sorafenib or BMS-1166 at a final concentration 0.5 mg/ml. A pre-sterilized membrane (pore size = 0.22 μm) was used for filtration. Then, pre-sterilized 10-ml centrifuge tubes were used for aliquoting. Drugs were stored at −80°C. During experiments, drugs were stored at 4°C and protected from light.

#### 2.7.3 Nuclide Uptake Experiments (Feng et al., 2018b; Shao et al., 2018)

MHCC97-H cells were inoculated into SCID mice to enable formation of subcutaneous tumor tissue. Then, SCID mice were administered (by oral gavage) the same dose (0.2 g/kg) of a lipid emulsion or SNM once-daily three times over 3 days. After that, mice were transferred to a small-animal anesthesia machine for inhalation anesthesia. The induction of anesthesia was with 3.5% isoflurane (*v*/*v*), and anesthesia was maintained with 1.5% isoflurane (*v*/*v*).

Mice were removed from the small-animal anesthesia machine, and a radionuclide probe (^18^F-fluorodeoxyglucose (^18^F-FDG)) was injected at 200 μCi (i.e., 7.4 MBq) into the tail vein. Mice were allowed at rest for 30–40 min after injection. Then, tumors were collected. The nuclide intensity in tissue and blood was quantified using a Geiger counter. The specific method of Nuclide intensity detection is: separately detected the nuclide intensity in a unit weight of blood or HCC tissue samples, and taken the blood sample as the unit 1 to calculate the multiple folds of the tissue relative to the blood [i.e., folds of blood].

#### 2.7.4 Creation of a Subcutaneous Tumor Model (Feng et al., 2019; Sun et al., 2019)

Cells were prepared as suspensions using sterilized PBS or physiologic (0.9%) saline. Cell suspensions were injected (s.c.) into the medial femoral vein of the lower limbs of SCID mice. About 5 × 10^6^ HCC cells were injected per point. After inoculation, the eating status, mental state, and whether the skin at the inoculation site was ulcerated were observed each day. About 2–3 days after inoculation, the injected volume had been absorbed. At this time, drug treatment was carried out for 2–3 weeks.

After inoculating SCID mice with MHCC97-H cells subcutaneously, they were placed in four groups randomly and treated with: 1) the SNM administered by oral gavage every other day for 3 weeks; 2) the SNM and sorafenib by oral gavage every other day for 3 weeks; 3) the SNM and BMS-1166 by oral gavage every other day for 3 weeks; 4) PBLs through tail-vein injection. Approximately 1 × 10^6^ cells were administered to SCID mice each time. Cells were re-infused every 3 days.

After subcutaneous inoculation of BALb/c mice with H22 cells, the SNM sorafenib, or BMS-1166 were administered by oral gavage, respectively, every other day for 3 weeks. The volume and weight of tissue were measured to determine the antitumor activity of the drug. For measurement of tumor volume, the length (i.e., long axis/length) and width (W/i.e., short axis) of subcutaneous tumor tissue were measured precisely using a vernier caliper. Then, the formula length × W × W/2 was used to calculate the volume of the subcutaneous tumor (mm^3^). A precision balance (1/10,000) was used to weigh subcutaneous tumor tissue.

Cells were passed through a 200-mesh steel sieve. Then, cells were resuspended into a single-cell suspension, and washed with Dulbecco’s modified Eagle’s medium containing 20% fetal bovine serum. The suspension of tumor tissue was sorted on a flow cytometer, and the human CD45^+^ component was used as human peripheral blood lymphocytes and the trypan blue was used to ascertain if cells had survived. The survival rates of PBLs in HCC tumor tissues were calculated according to the PBLs number, survival PBLs number in the resuspended cell suspension from tumor tissue corresponds to 50 mg.

#### 2.7.5 Biochemical Detection of Subcutaneous Tumor Tissue and Cell Samples

For cell samples, steel balls were employed for lysing. For subcutaneous tumor tissue, liquid nitrogen was employed for grinding. Ground tissue was weighed, PBS was added (200 μl of PBS per 100 mg of tissue), followed by thorough mixing. Centrifugation was carried out (12,000 rpm, 15 min, 4°C), after which the supernatant was collected for biochemical analyses.

In HCC tissues, we used colorimetric kits to measure the level of glucose (ab136955; Abcam, Cambridge, United Kingdom), lactate (Lactate-Glo™; Promega, Fitchburg, WI, United States), adenosine triphosphate (ATP) (ab83355; Abcam), and lactate dehydrogenase (LDH; MAK066; MilliporeSigma, Burlington, MA, United States). For cell samples, sonication was undertaken, followed by biochemical assays (Li et al., 2017b; Li et al., 2018b). Levels of these metabolism-related factors were compared with those of the control group. On this basis, heatmaps were drawn according to the relative expression of each target gene, as described above (Ma et al., 2020; Zhou et al., 2021).

### 2.8 Statistical Analyses

Statistical analyses were carried out using the Bonferroni correction with two-way ANOVA and paired-sample *t*-tests. SPSS 9.0 (IBM, Armonk, NY, United States) was used for statistical analyses. Half-maximal inhibition (IC_50_) values of antitumor dugs were calculated using Origin 6.1 (OriginLab, Northampton, MA, United States).

## 3 Results

### 3.1 The SNM Did Not Affect the Metabolic Profile of HCC Cells *In Vivo* Compared With That of a Lipid Emulsion

**FIGURE 1:**
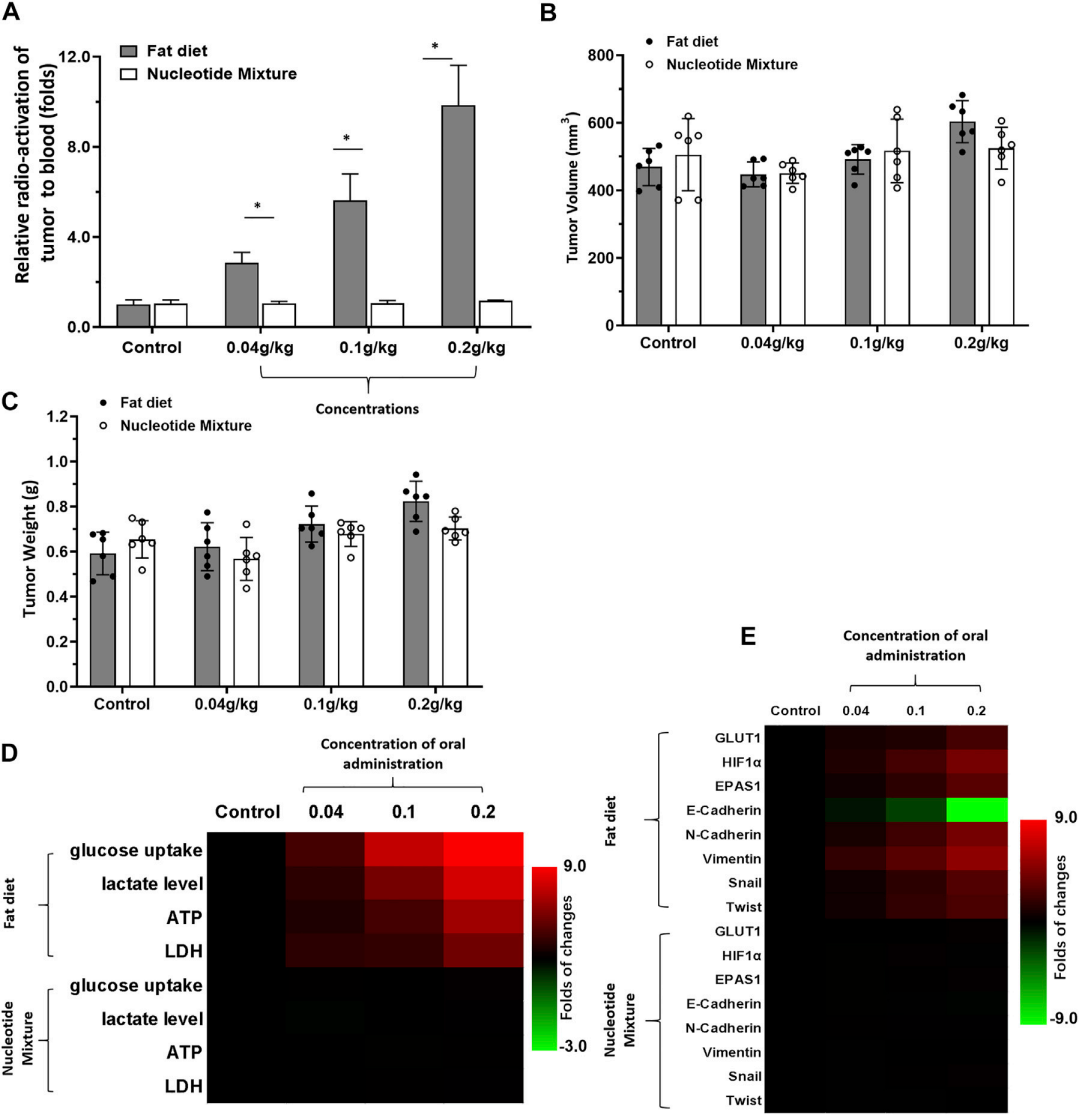
Detection of the metabolic status of MHCC97-H cells in SCID mice by a lipid emulsion and the SNM. MHCC97-H cells were obtained by culture. Then, cells were inoculated into SCID mice subcutaneously. After the formation of tumor tissue, the Nuclide uptake experiments was done on SCID mice. The nuclide intensity of HCC tumor tissues folds to blood was shown as Histogram **(A)**. Tumor tissues were collected and the tumor volume **(B)** and tumor weight **(C)** shown as histograms. The level of metabolism-related indicators **(D)** and Warburg effect-related factors **(E)** in tumor tissues are shown as heatmaps. *p < 0.05.

First, the effects of the SNM with the lipid fat emulsion containing nutritional supplements was undertaken (Figure 1). Upon short-term administration, the lipid emulsion (0.2 g/kg) affected the metabolic characteristics of MHCC97-H cells in SCID mice significantly. Uptake of glucose and abnormal metabolism were enhanced significantly. This phenomenon was reflected in ^18^F-FDG uptake by MHCC97-H cells being significantly higher than that of the control group (Figure 1A). Short-term intake of the lipid emulsion or SNM did not affect the volume or weight of the tumor (Figures 1B,C). Levels of glucose, lactate, ATP, and LDH in the lipid-emulsion group were significantly higher than those of the control group (Figure 1D). The SNM administered orally did not affect the metabolic characteristics of MHCC97-H cells in SCID mice: MHCC97-H cells in the SNM group had no effect on ^18^F-FDG uptake (Figure 1A). Levels of glucose, lactate, ATP, or LDH were not significantly different in the SNM group compared with those of the control group (Figure 1D). RT-qPCR was undertaken to ascertain the Warburg effect and epithelial–mesenchymal transition (EMT)-related factors in tumor tissues (Figure 1E). Oral administration of the lipid emulsion (but not the SNM) could induce expression of Warburg effect-related factors in MHCC97-H cells and induce an EMT effect in cells. We documented upregulation of expression of the Warburg effect-related factors glucose transporter 1 (GLUT1), endothelial PAS domain-containing protein 1 (EPAS-1), and hypoxia inducible factor 1 (HIF-1α) (Figure 1E), upregulated expression of the EMT-related factors N-Cadherin, vimentin, Twist and Snail (Figure 1E), and downregulated expression of the epithelial marker E-Cadherin (Figure 1E).

### 3.2 The SNM Could Promote the Proliferation of MHCC97-H Cells in SCID Mice to a Certain Extent

**FIGURE 2:**
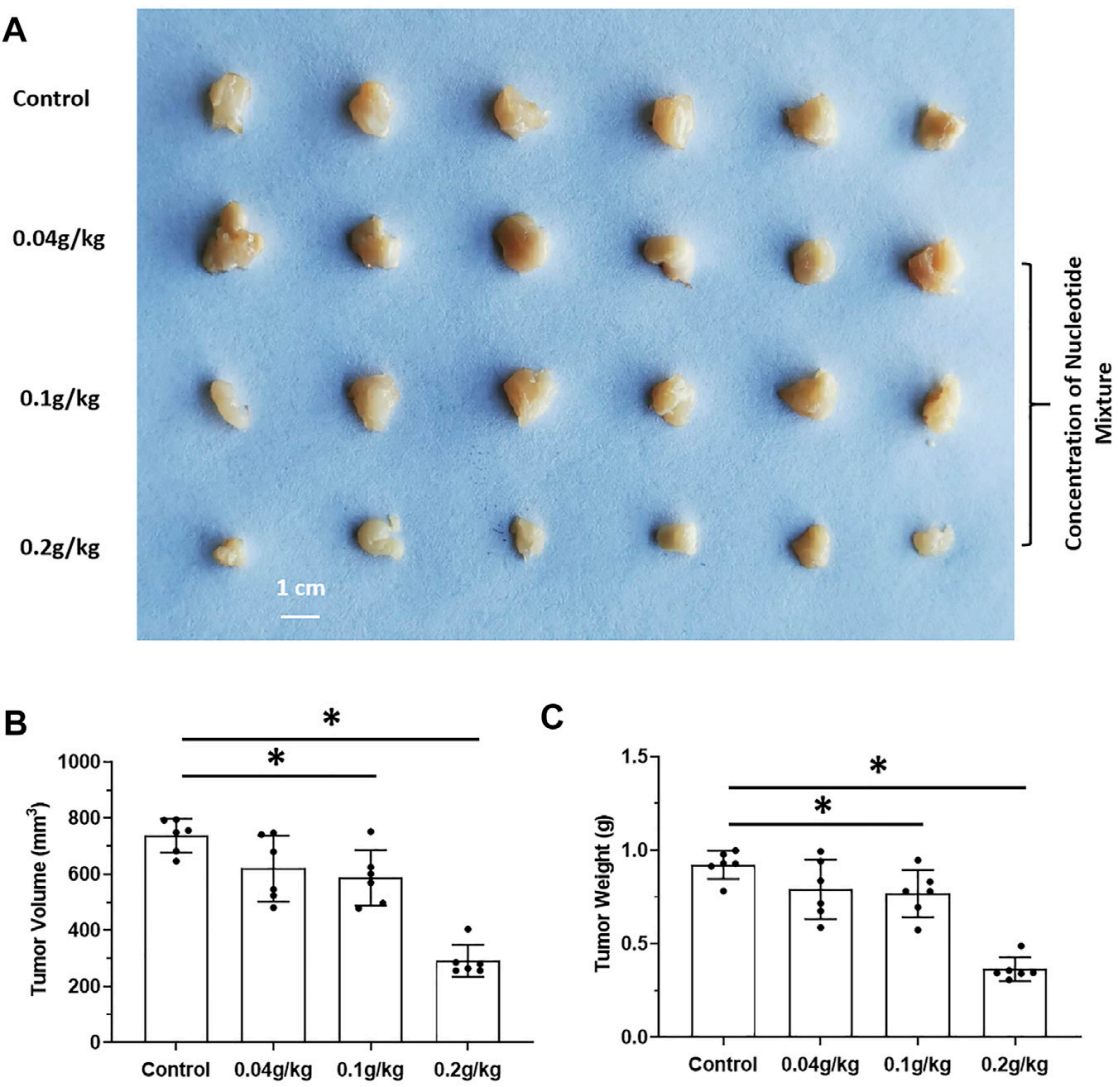
The tumorigenic effect of nucleotide mixture alone on mouse HCC cells H22 in immunized normal mice. H22 cells were inoculated into BalB/c mice subcutaneously. Then, mice were administered (p.o.) with the SNM (0.04, 0.1, 0.2 g/kg). A photograph of a subcutaneous tumor **(A)** and the tumor volume **(B)**, tumor weights **(C)** are shown. *p < 0.05.

**FIGURE 3:**
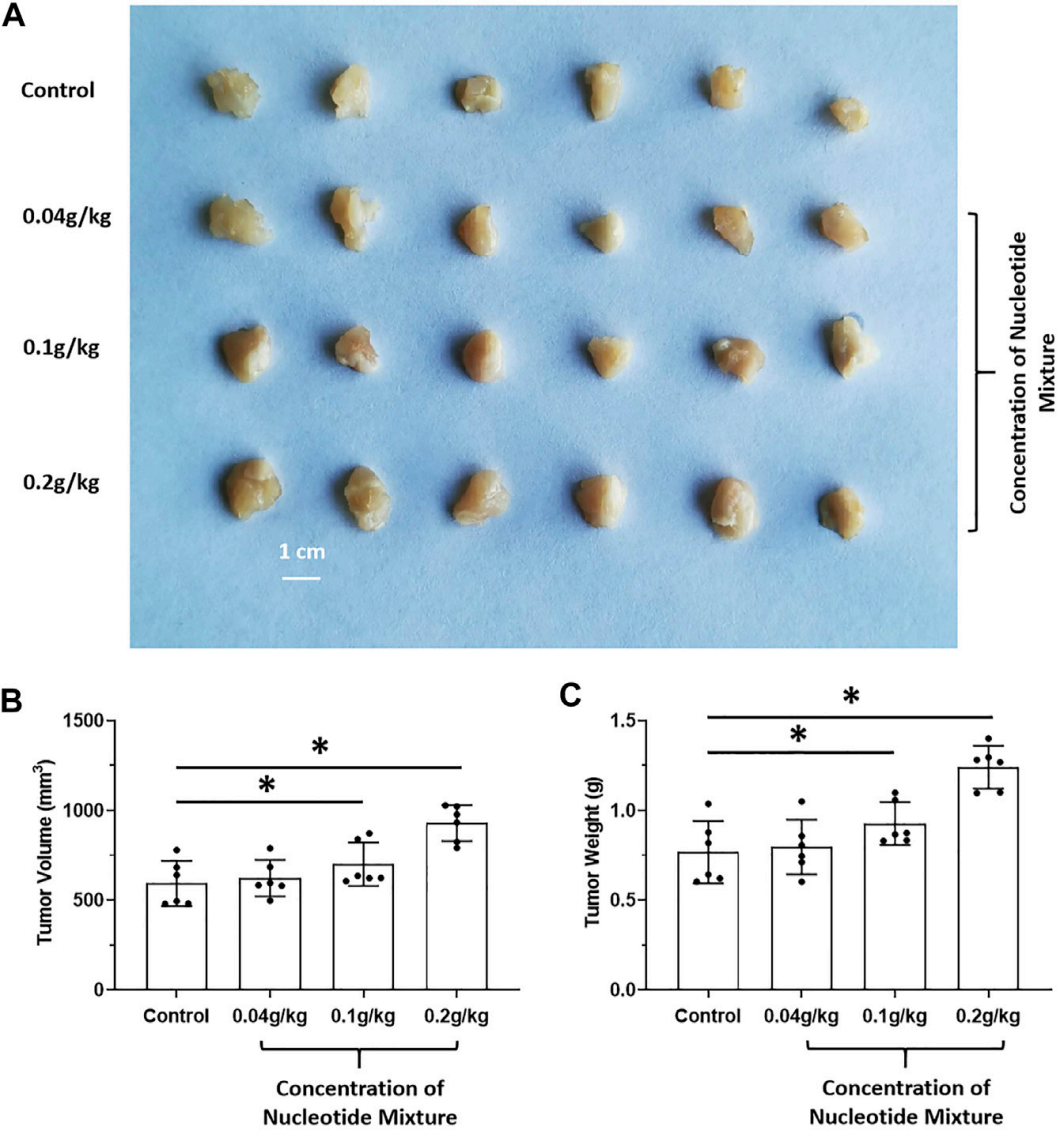
Tumorigenic effect of the SNM alone on human HCC cells in immunodeficient mice. MHCC97-H cells were obtained by culture. Then, cells were inoculated into SCID mice subcutaneously. Next, mice were administered (p.o.) the SNM (0.04, 0.1, 0.2 g/kg). A photograph of a subcutaneous tumor **(A)** and the tumor volume **(B)** tumor weights **(C)** are shown. *p < 0.05.

The effects of long-term administration of the SNM on proliferation of human MHCC97-H cells in SCID mice and proliferation of murine H22 cells in BalB/c mice were detected. The SNM had different effects on H22 cells and MHCC97-H cells. The SNM could slow down the growth of H22 cells in BalB/c mice in a dose-dependent manner, and the high-dose group (0.2 g/kg) had the most obvious anti-tumor effect (Figure 2). The SNM had no clear anti-tumor activity on MHCC97-H cells in SCID mice, and the high-dose group (0.2 g/kg) may have elicited a tumor growth-promoting effect (Figure 3). These data suggested that the antitumor activity of the SNM was related to the species characteristics of cells in experimental mice.

### 3.3 The SNM Could Have a Protective Effect Upon Immune Cells

**FIGURE 4:**
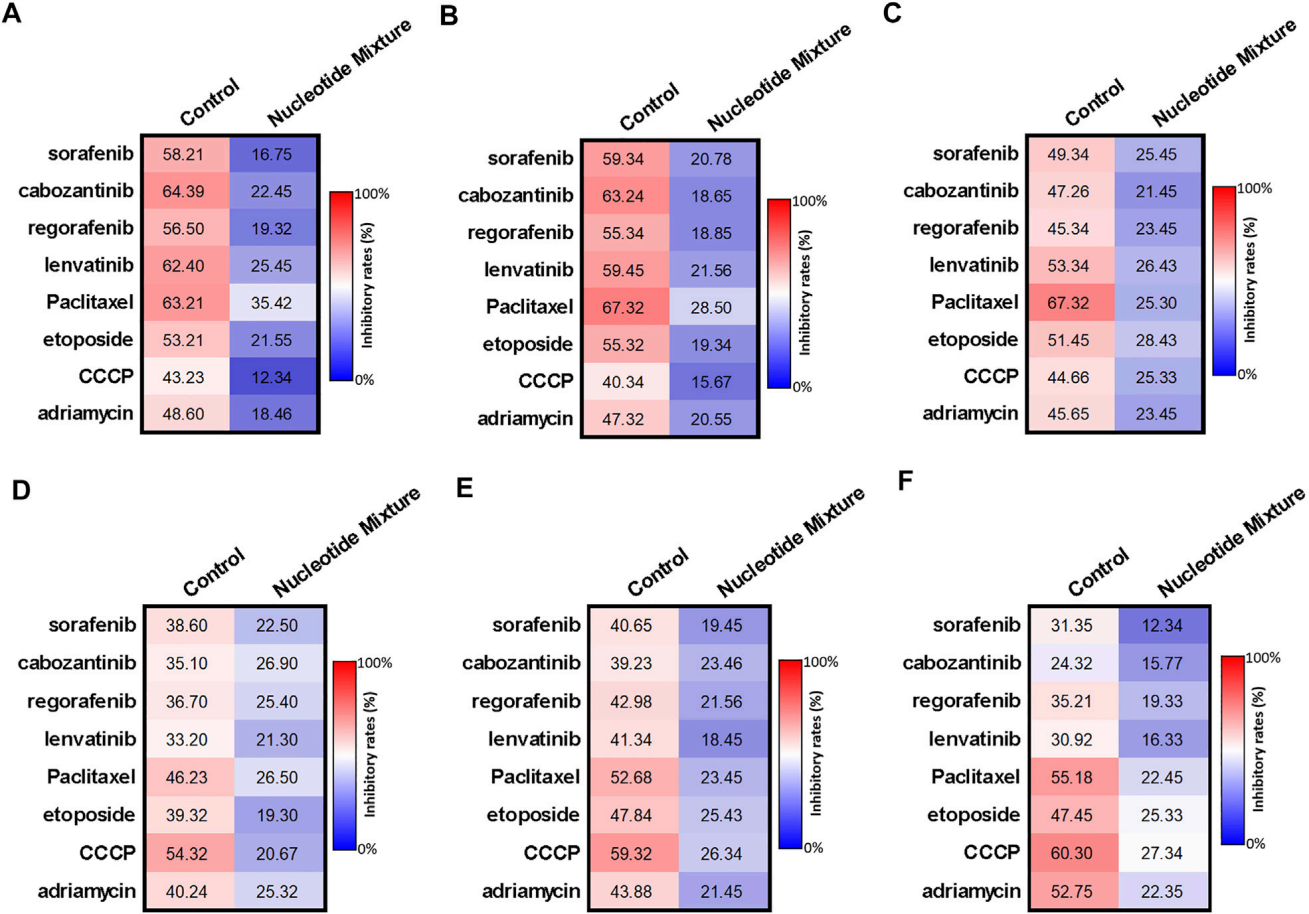
Protective effect of the SNM on cells isolated from the peripheral blood lymphocytes of healthy volunteers. Peripheral-blood lymphocytes were isolated from blood donated from healthy volunteers. T cells **(A,D)**, B cells **(B,E)**, and NK cells **(C,F)** were obtained by sorting. T cells **(A,D)**, B cells **(B,E)**, or NK cells **(C,F)** were treated with immunotoxic drugs (sorafenib, cabozantinib, regorafenib, and lenvatinib at 1 μmol/L; paclitaxel (300 nmol/L), etoposide, (300 nmol/L), CCCP ((300 nmol/L), and adriamycin (100 nmol/L) alone or in combination with the SNM. Then, the MTT assay **(A–C)**, or apoptosis **(D–F)** assay were conducted to determine the damage wrought by these drugs on T cells **(A,D)**, B cells **(B,E)**, or NK cells **(C,F)**. The results are shown as heatmaps.

**FIGURE 5:**
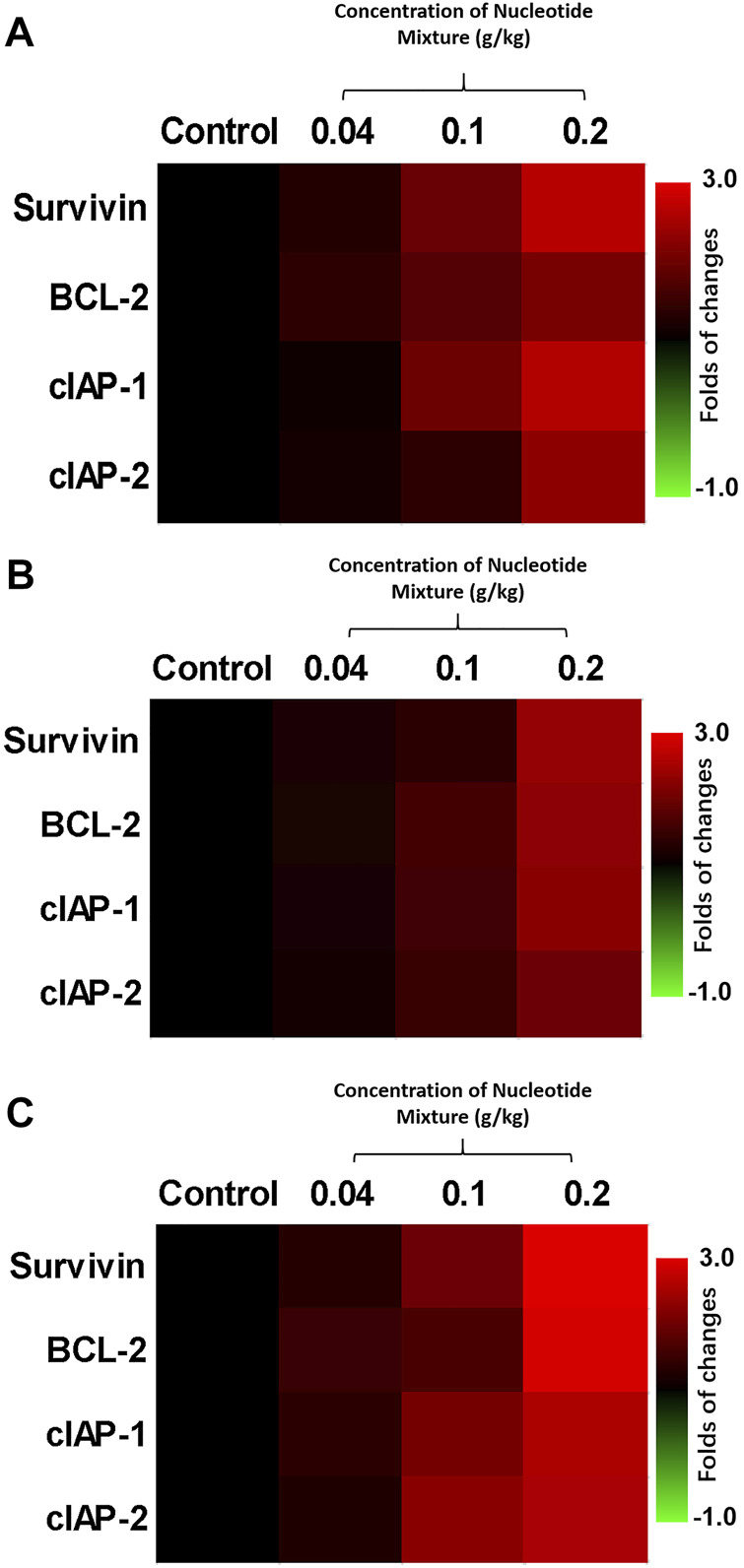
Protective effect of the SNM on cells isolated from the peripheral-blood lymphocytes of healthy volunteers (RT-qPCR). Peripheral-blood lymphocytes were isolated from blood donated from healthy volunteers. Then, T cells **(A)**, B cells **(B)**, and NK cells **(C)** were obtained by sorting. Cells were treated with different doses of the SNM, and the effects on expression of BCL-2, survivin, cIAP-1, and cIAP-2 in cells were examined by RT-qPCR. Results are shown as heatmaps of the fold change in expression of pro-survival and anti-apoptotic factors in each group compared with the control group.

Use of H22 cells and MHCC97-H cells showed that the difference in the anti-tumor activity of the SNM was due mainly to the immune status of experimental mice. Hence, the effect of the SNM on immune cells was examined (Figure 4). Several MTDs (sorafenib, cabozantinib, regorafenib, lenvatinib) and cytotoxic chemotherapy drugs in the nmol/L range (paclitaxel (30 nmol/L), etoposide (300), etoposide (300), CCCP (300), adriamycin (100)) could damage lymphocytes (T cells (Figures 4A,D), B cells (Figures 4B,E), and NK cells (Figures 4C,F) derived from the peripheral blood of HVs. The SNM could exert a protective effect on these three types of lymphocytes, and significantly downregulated the effects of these drugs on lymphocytes (Figure 4). Similar results were obtained from the assays on cell survival (MTT) (Figures 4A–C) and apoptosis (Figures 4D–F). Simultaneously, RT-qPCR was used (Figures 5A–C). The SNM could upregulate expression of the pro-survival and anti-apoptosis-related factors B-cell lymphoma 2 (BCL-2), survivin, cIAP1 (cellular inhibitor of apoptosis 1), and cIAP2 (cellular inhibitor of apoptosis 2) in T cells (Figure 5A), B cells (Figure 5B), and NK cells (Figure 5C). These data suggested that the SNM could exert a protective effect upon immune cells.

### 3.4 The SNM Could Promote the Recruitment and Survival of Immune Cells in Tumor Tissue

**FIGURE 6:**
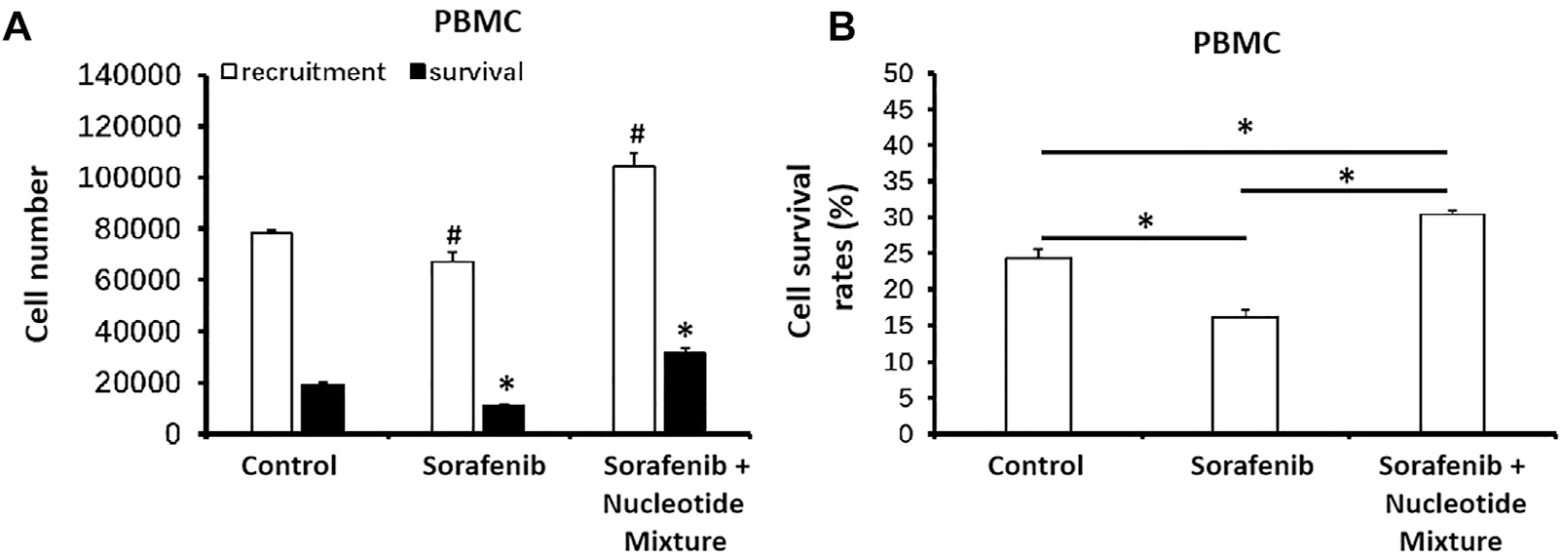
The SNM could promote the recruitment and survival of healthy volunteer-derived peripheral-blood lymphocytes in the subcutaneous tumors formed by MHCC97-H cells in mice. MHCC97-H cells were obtained by culture. Then, cells were inoculated subcutaneously into SCID mice to enable formation of tumor tissue. Simultaneously, peripheral-blood lymphocytes from healthy volunteers were infused back into SCID mice (∼1 × 10^7^ cells per mouse). Then, mice received sorafenib (1 mg/kg, p.o.) or a combination of sorafenib (1 mg/kg) + SNM (0.2 g/kg). Then, tumor tissue was collected, ground, and flow cytometry-sorted to determine the level in each 50 mg of tissue. The number of peripheral-blood lymphocytes recruited (CD45^+^ fraction) and the number of surviving peripheral-blood lymphocytes (CD45 combined with trypan-blue staining) **(A)** or survival of cells **(B)** are shown as histograms. **(A)** *p < 0.05 compared with control (recruitment of peripheral-blood lymphocytes); **(B)** #*p* < 0.05 compared with control (survival of peripheral-blood lymphocytes). *p < 0.05.

Next, MHCC97-H cells were inoculated into SCID mice and then infused with human PBLs. Sorafenib monotherapy could damage lymphocytes, thereby resulting in a decreased distribution of lymphocytes in the tumor tissue formed by MHCC97-H cells (Figure 6A). Combination of sorafenib with the SNM enabled the latter to significantly reduce the damage wrought by sorafenib on lymphocytes, and promote PBL recruitment in the subcutaneous tumor tissue formed by MHCC97-H cells (Figure 6A). Simultaneously, the SNM could significantly improve lymphocyte survival in the tumor tissue formed by MHCC97-H cells (Figure 6B).

### 3.5 The SNM Could Achieve Effective Anti-Tumor Activity After Humanized Immune Reconstitution

**FIGURE 7:**
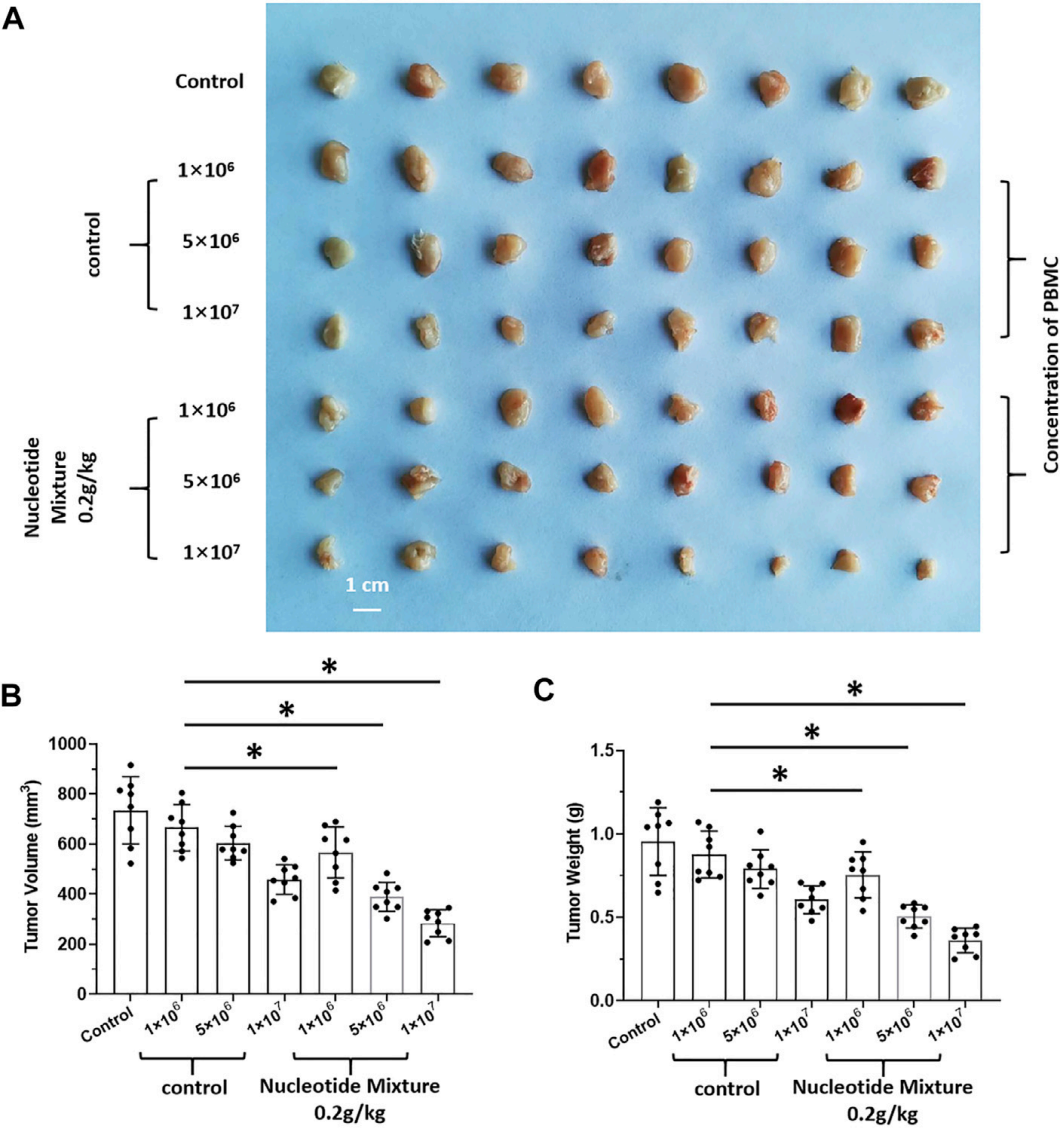
Combined infusion of nucleotide mixture and healthy human peripheral blood lymphocytes on tumorigenesis of human HCC MHCC97-H in immunodeficient mice. MHCC97-H cells were obtained by culture. After MHCC97-H cells had been inoculated subcutaneously into SCID mice, peripheral-blood lymphocytes were isolated from human volunteers. SCID mice were inoculated with 1 × 10^6^, 5 × 10^6^, or 1 × 10^7^ cells each time/mouse (once every 2 days, 10 times in total). During injection of peripheral-blood lymphocytes, mice received the control or SNM (0.2 g/kg) every day. After treatment, tumor tissue was collected and photographed **(A)**. Photographs of subcutaneous tumor tissue **(A)**, tumor volume **(B)**, and tumor weight **(C)**. *p < 0.5.

**FIGURE 8:**
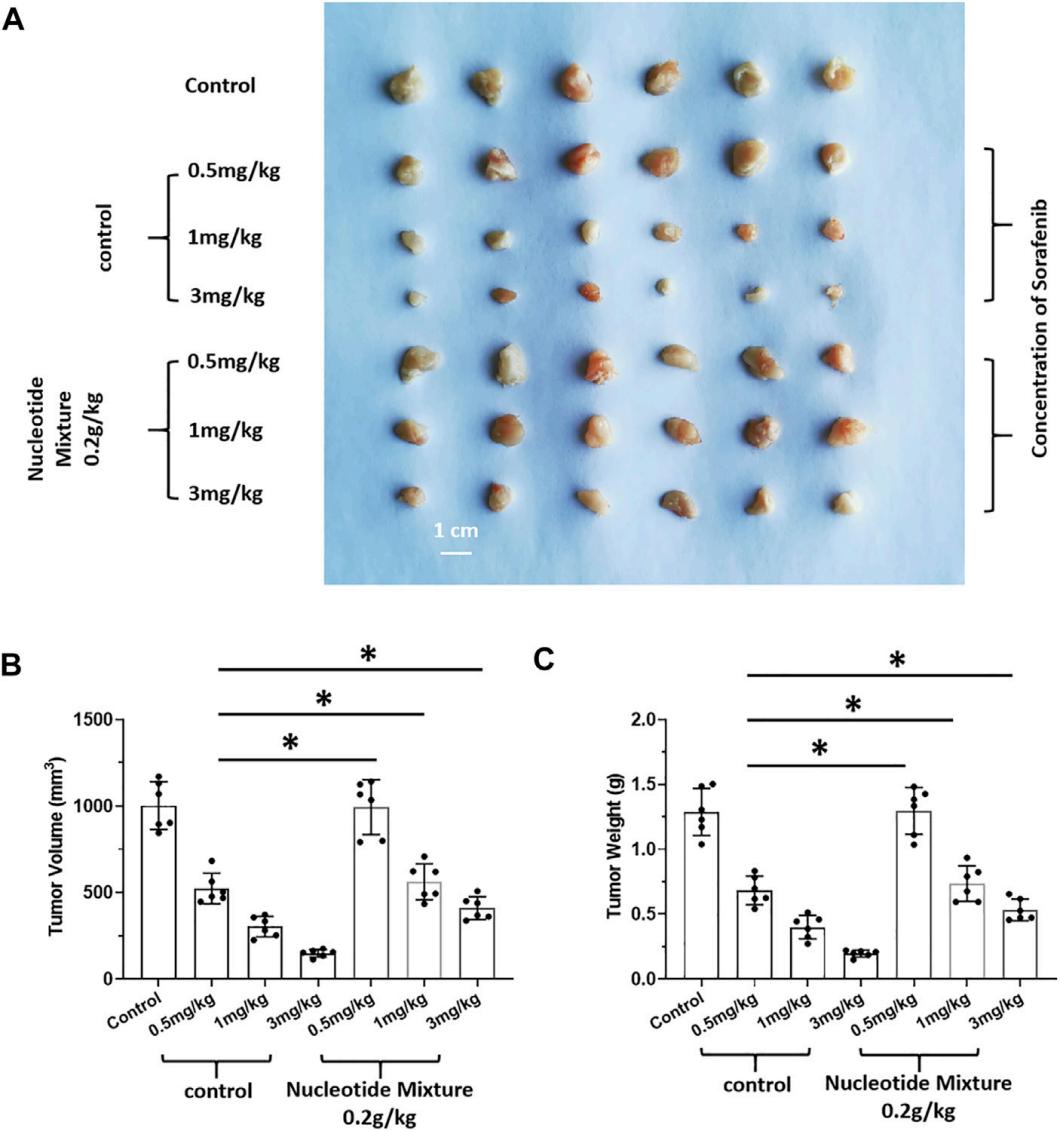
Effect of the SNM on molecular-targeted drugs inhibiting the subcutaneous growth of human HCC cells in immunodeficient mice. MHCC97-H cells were obtained by culture, and inoculated subcutaneously in immunodeficient SCID mice. Then, mice were given sorafenib (0.5, 1, 3 mg/kg) alone or combined with the SNM (0.2 g/kg). Photographs of subcutaneous tumors **(A)**, tumor volume **(B)**, or tumor weight **(C)** are shown. *p < 0.05.

**FIGURE 9:**
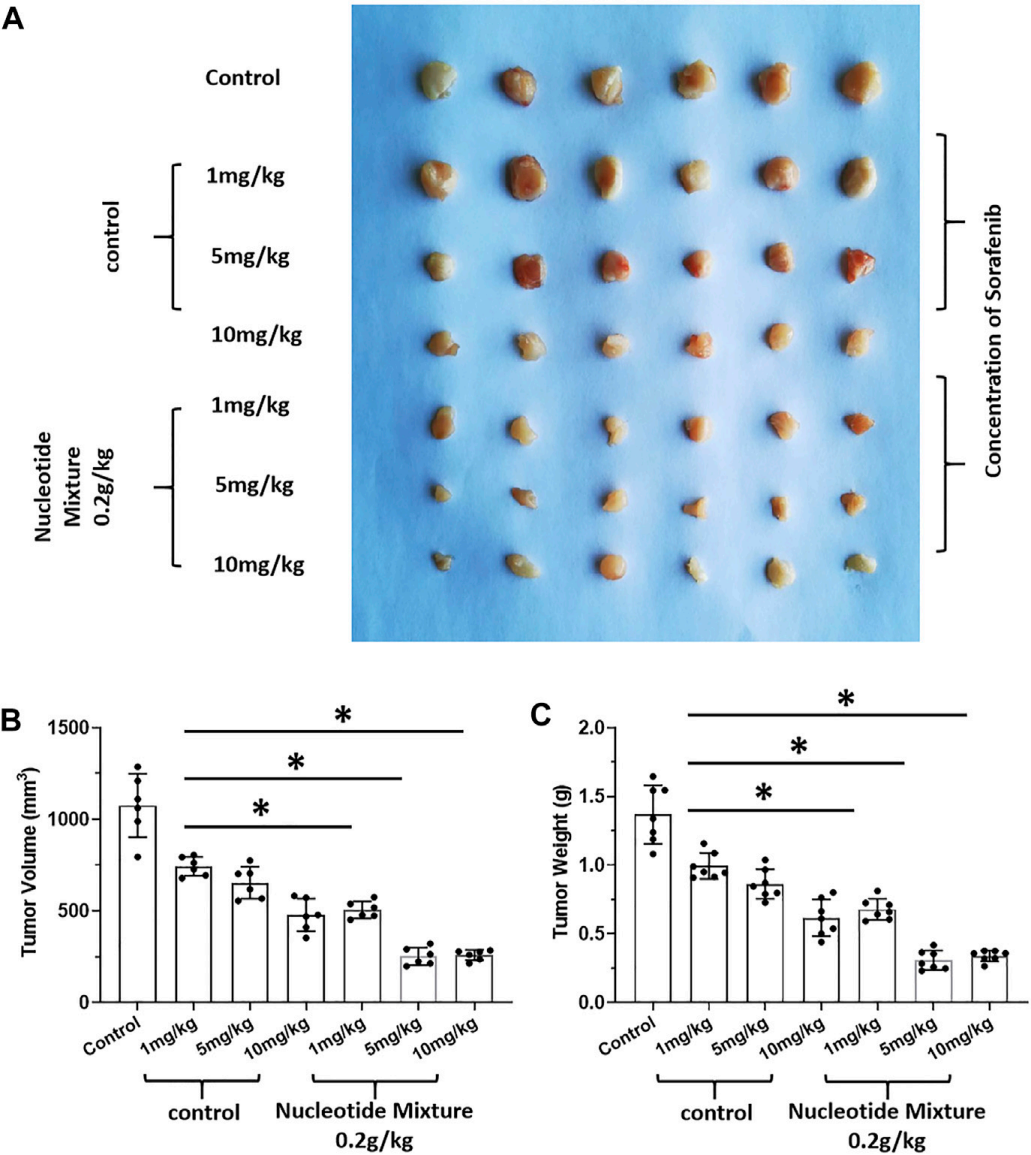
Effect of the SNM on molecular-targeted drugs inhibiting the subcutaneous growth of murine HCC cells in BalB/c mice. H22 cells were obtained and inoculated subcutaneously in BalB/c mice. Then, mice were given sorafenib (1, 5, 10 mg/kg) alone or combined with the SNM (0.2 g/kg). Photographs of subcutaneous tumors **(A)**, tumor volume **(B)**, and tumor weight **(C)** are shown. *p < 0.05.

After SCID mice had been inoculated (s.c.) with human the HCC line MHCC97-H, they were given infusions of PBLs from HVs at low (1 × 10^6^), medium (5 × 10^6^), and high (1 × 10^7^) doses. PBLs had a certain anti-tumor activity and could diminish the tumorigenic effect of MHCC97-H in nude mice dose-dependently (Figure 7). Simultaneously, compared with the control group, the SNM could improve the anti-tumor activity of PBLs significantly (Figure 7). Sorafenib could inhibit the tumorigenic effect of MHCC97-H cells in SCID mice dose-dependently, but the SNM (0.2 g/kg) repressed the antitumor effect of sorafenib (Figure 8). Different from these results, sorafenib inhibited the subcutaneous growth of H22 cells in a dose-dependent manner, and the SNM could improve the anti-tumor activity of sorafenib significantly (Figure 9). We wished to confirm the effect of the SNM on TKIs or ICIs. H22 cells were inoculated into BalB/c mice, and MHCC97-H cells were inoculated into SCID mice. SCID mice were infused with PBLs from HVs (1 × 10^7^ cells each time, and infused thrice). The six doses of TKI alone or ICI alone or in combination with the SNM were given to mice. The SNM could improve the anti-tumor activity of sorafenib or BMS-1166 significantly, and the IC_50_ of sorafenib and BMS-1166 in tumor tissue or the tumor weight decreased, respectively (Table 3).

**TABLE 3:** The single nucleotide mixture can down-regulate the *IC*_*50*_ value of a series of concentrations of sorafenib or BMS-1166 in HCC cells.

Cell lines	Groups	Sorafenib	BMS-1166
		*IC*_*50*_ values (mg/kg)	
MHCC97-H	tumor volumes	0.52 ± 0.27	2.59 ± 0.50
	tumor weights	0.11 ± 0.05	0.75 ± 0.38
H22	tumor volumes	3.84 ± 0.79	5.66 ± 0.67
	tumor weights	1.04 ± 0.61	0.64 ± 0.48

Table notes: H22 cells were still inoculated into BalB/c mice, and after MHCC97-H cells were inoculated into SCID mice, the SCID mice were infused with healthy human-derived peripheral blood lymphocytes (1 × 10^7^ cells for each time and perfumed time times). The concentrations of sorafenib: 3, 2, 1, 0.5, 0.2, 0.1 mg/kg in SCID mice and 10, 5, 2, 1, 0.5, 0.2 mg/kg in BalB/c mice; BMS-1166: 10, 5, 2, 1, 0.5, 0.2 mg/kg in SCID mice and 20, 10, 5, 2, 1, 0.5 mg/kg in BalB/c mice.

## 4 Discussion

TKIs were once the only drug treatment for advanced HCC (Roskoski, 2019; Roskoski, 2020; Roskoski, 2021; Roskoski, 2022). These TKIs (e.g., sorafenib) can delay HCC progression and prolong patient survival (Zhu et al., 2017), but they face three main challenges. First, the vast majority of patients who are initially sensitive to sorafenib develop drug tolerance as treatment progresses (Zhu et al., 2017). Second, existing treatment strategies (e.g., sorafenib, 800 mg, p.o.) can elicit serious side-effects (Zhu et al., 2017). Lenvatinib has been approved as a first-line (Bruix et al., 2017; Kudo et al., 2018) and regorafenib as a second-line (Feng et al., 2018a; Zongyi and Xiaowu, 2020) drug, and they are considered superior to sorafenib. However, they are chemically similar and all share the same parent nucleus [1-(4-(pyridin-4-yloxy) phenyl)urea] (He et al., 2021b; Jiang et al., 2021). Hence, overcoming the problems of sorafenib treatment is difficult. Different from existing research, we focused on the influence of the nutritional status and immune status of mice on treatment effects. The existing strategies for nutritional support for HCC patients are: 1) providing energy support (mainly an intravenous infusion of glucose); 2) oral, nasogastric, or injection routes of high-energy-density lipid emulsions as nutritional supplements; 3) Chinese patent medicines (Zhang et al., 2017b). A lipid emulsion was used as a control for the SNM in our study. Administration of a lipid emulsion by oral gavage could induce the Warburg effect (Hu et al., 2021; Yi et al., 2022), expression of Warburg effect-related factors in HCC cells, and promote expression of various proliferation- and survival-related factors of malignant tumor cells in experimental mice. In contrast, the SNM did not induce the Warburg effect or expression of various proliferation- and survival-related factors in HCC cells. First, MHCC97-H cells were inoculated into SCID mice. Short-term administration of a drug was undertaken after MHCC97-H cells formed tumor tissues in SCID mice (administration of the lipid emulsion or SNM once-daily for three consecutive days by oral gavage). The drug did not affect the volume of HCC tumor tissue significantly within a 3-day dosing cycle. The RT-qPCR and other assays could intuitively reflect the direct effects of the SNM on HCC cells. We chose a lipid emulsion instead of glucose because the effect of an oral solution or injection of glucose on the metabolism and proliferation of HCC cells is well known: abnormal glucose metabolism is an important feature of malignant tumor cells as represented by HCC cells (Bose et al., 2021; Vaupel and Multhoff, 2021; Wiese et al., 2021). Glucose administration to experimental animals affects the metabolic characteristics of HCC tissues and expression of proliferation-related factors (Broadfield et al., 2021; Loong et al., 2021). Cellular metabolism of glucose and lipids is closely related, and important regulators such as the transcription factors sterol regulatory element-binding transcription factor 1 (SREBP-1) and SREBP-2 can also affect expression of glucose metabolism-related factors and cellular glucose metabolism (Yin et al., 2019; Zou et al., 2021). Therefore, our results suggest that glucose (energy support) and a lipid emulsion (nutritional support) will improve the physical status of patients, but may also promote HCC *in vivo*. Hence, a SNM may be preferable.

Immunotoxicity is the main and non-negligible side-effect of various anti-tumor treatment strategies (Buoso et al., 2021; Shanti et al., 2021). Upon anti-tumor treatment, the number of lymphocytes (especially in peripheral blood) drops sharply in patients (Fessas et al., 2020; Wesley et al., 2021). The adjuvant drugs employed for treatment of early HCC are interferons (e.g., gamma-interferon), interleukin-2, and immunomodulators such as albumin or thymosin (Ding et al., 2018; Yan et al., 2021). In addition, a considerable proportion of patients also receive albumin injections. However, such strategies do not: alleviate the damage to the immune system caused by anti-tumor treatment strategies; exert a protective effect upon lymphocytes; help to improve the function of the immune system; protect immune-related stem cells or blast cells. In addition, patients with advanced HCC are often accompanied by different degrees of fibrosis, cirrhosis, functional insufficiency of the liver or portal hypertension (Behary et al., 2021; Mohr et al., 2021; Raza et al., 2021). Therefore, patients need continuous infusion of albumin and immune enhancement to prevent infection.

The SNM we used is different from immune-related cytokines or albumin. As an important low-molecular-weight compound in an organism, the SNM comprises three main components: base, pentose, and phosphate. These components are the basic units of nucleic acids. The latter determine the biological characteristics, proteins, and functions of cells, and control the growth, development, reproduction, and inheritance of organisms (Cai et al., 2016a; Cai et al., 2016b). Nucleotides also participate in regulation of the metabolism of various substances and of various protein functions in the form of free nucleotides or their derivatives (Cai et al., 2016a; Cai et al., 2016b). Free nucleotides are the main high-energy compounds in energy-metabolism pathways, important messengers in signal transduction within cells, and metabolic regulators of various nutrients in the body (Cai et al., 2016a; Cai et al., 2016b). Nucleotides are synthesized and degraded continuously in organisms, and there are two main sources, i.e., endogenous nucleotides: 1) present in the body; 2) synthesized by enzymatic hydrolysis and other technologies (Cai et al., 2016a; Cai et al., 2016b). Nucleotides from exogenous sources (e.g., DNA, RNA nucleotides/nucleosides) are indispensable nutrients under specific physiological conditions (Cai et al., 2016a; Cai et al., 2016b). Studies have shown that addition of exogenous nucleotides can nourish lymphocytes, promote their differentiation, and inhibit DNA damage, thereby improving cellular immune function, humoral immune function, and monocyte–macrophage phagocytosis (Cai et al., 2016a; Cai et al., 2016b). Those observations are consistent with our findings. The SNM had strong antitumor activity (or adjuvant antitumor activity) in normal (but not immunodeficient) mice. After re-immunization in immunodeficient mice, the SNM regained antitumor activity. Simultaneously, we clarified the protective effects of the SNM on immune cells at multiple levels, all of which highlighted the advantages of the SNM over other immunomodulators.

Immunotherapy of HCC has been a major breakthrough in recent years. Immunotherapy targets PD-1 and its ligand (PD-L1) and other immune checkpoints (Llovet et al., 2022). MTDs, including therapeutic monoclonal antibodies or small-molecule inhibitors, offer new hope for patients (Yan et al., 2020; Yi et al., 2021). The overall efficacy of PD-1 inhibitors alone in treatment of liver cancer is about 10–30%. Due to the memory function of the immune system, some patients achieve clinical cure (complete remission), whereas the combination of a TKI and other MTDs can have a synergistic therapeutic effect (Llovet et al., 2022). However, long-term use of ICIs can cause immune system-related side-effects, and severe immune-related inflammatory reactions will occur (Han et al., 2020).

In the present study, sorafenib was selected as a representative TKI, and BMS-1166 was selected as a representative ICI targeting PD-L1 (Shi et al., 2019; Mittal et al., 2021). By setting seven concentrations for sorafenib and BMS-1166, the IC_50_ of each drug in MHCC97-H cells could be determined. The SNM could significantly enhance the killing effect of sorafenib and BMS-1166 in the human HCC line MHCC97-H. Despite species differences, sorafenib and BMS-1166 could inhibit the proliferation of murine H22 cells, which was also observed for H22 cells after inoculation into BalB/c mice. We selected sorafenib because it is the most widely used TKI. We selected BMS-1166 because it and sorafenib are small-molecule compounds, which aided comparison of their effects. The ICIs used to treat HCC are mainly therapeutic antibodies (Chen et al., 2020; Finn et al., 2020; Gordan et al., 2020; Hu et al., 2020; Yau et al., 2020; Liu et al., 2021b; Cao et al., 2021; Kelley et al., 2021; Pinter et al., 2021; Xu et al., 2021). Whether BMS-1166 can be applied to HCC merits further investigation. SNMs, small-molecule inhibitors, and therapeutic antibodies targeting PD-1/PD-L1 via immune mechanisms have different characteristics. PD-1/PD-L1-targeting therapies slow down/blockade the “immune escape” of HCC cells (targeting T cells mainly), whereas the SNM could protect immune cells directly and improve the overall immunity of patients. During treatment for HCC, the damage to immune cells arises mainly from: 1) the toxicity of anti-tumor drugs; 2) tumor tissue not being conducive to the survival and function of immune cells; 3) HCC cells affecting immune cells directly through PD-1/PD-L1 (immune escape by inducing T-cell apoptosis). Therefore, PD-1/PD-L1 and TKIs combined with a SNM may be a more advantageous strategy in the future.

In conclusion, this study focused on single nucleotide mixtures as an adjuvant therapy strategy for HCC, which not only expands our knowledge in related fields, but also provides more options for patients.

## Data Availability

The original contributions presented in the study are included in the article/Supplementary Material, further inquiries can be directed to the corresponding authors.
