# Supplementary figures and images for: A Single Nucleotide Mixture Enhances the Antitumor Activity of Molecular-Targeted Drugs Against Hepatocellular Carcinoma
Source: Front Pharmacol. 2022 Jun 27;13:951831. doi: 10.3389/fphar.2022.951831 (PMC9271877; doi:10.3389/fphar.2022.951831)

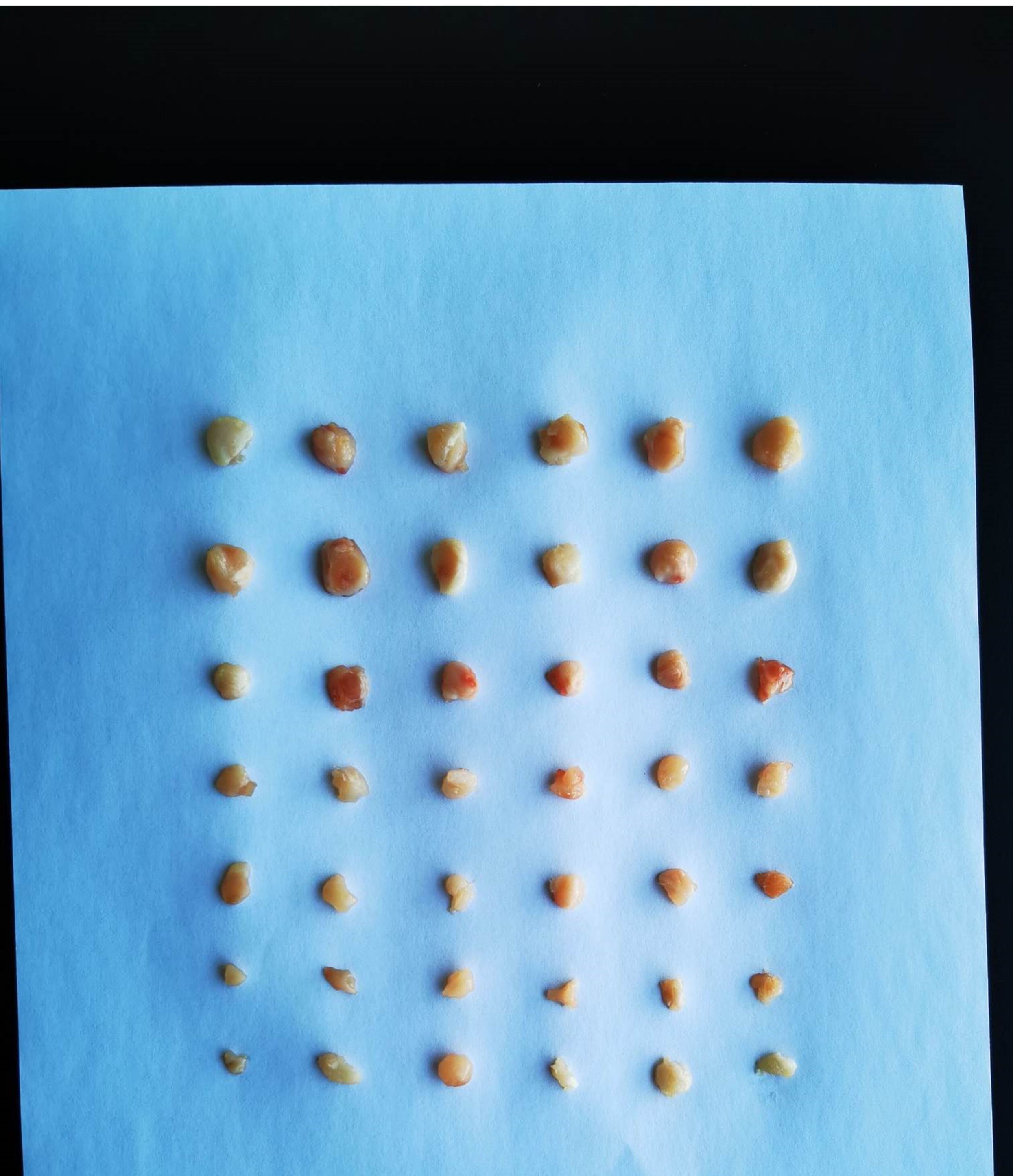

Supplement: Supplementary file 1 [file Image5.PNG]

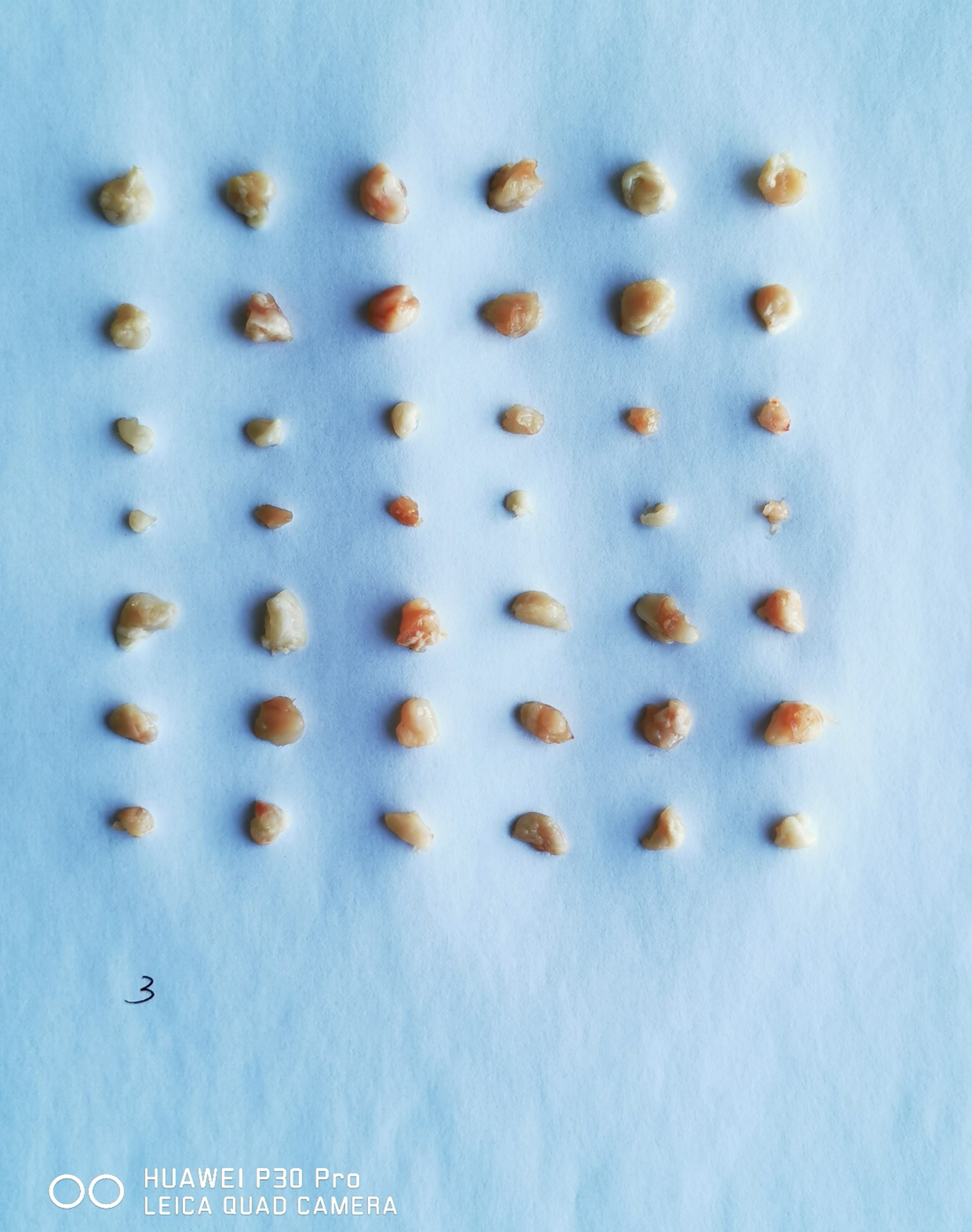

Supplement: Supplementary file 2 [file Image4.PNG]

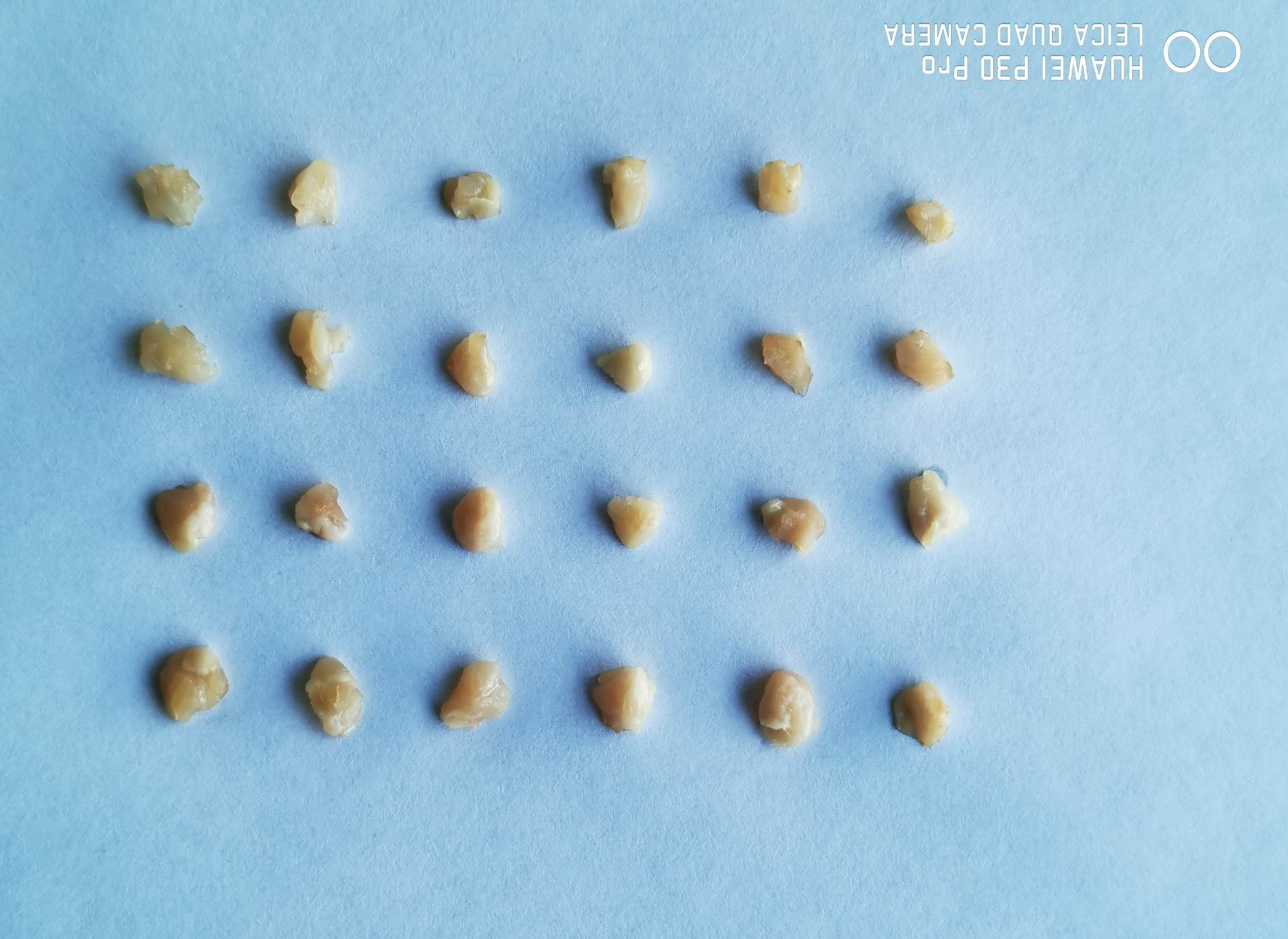

Supplement: Supplementary file 3 [file Image2.PNG]

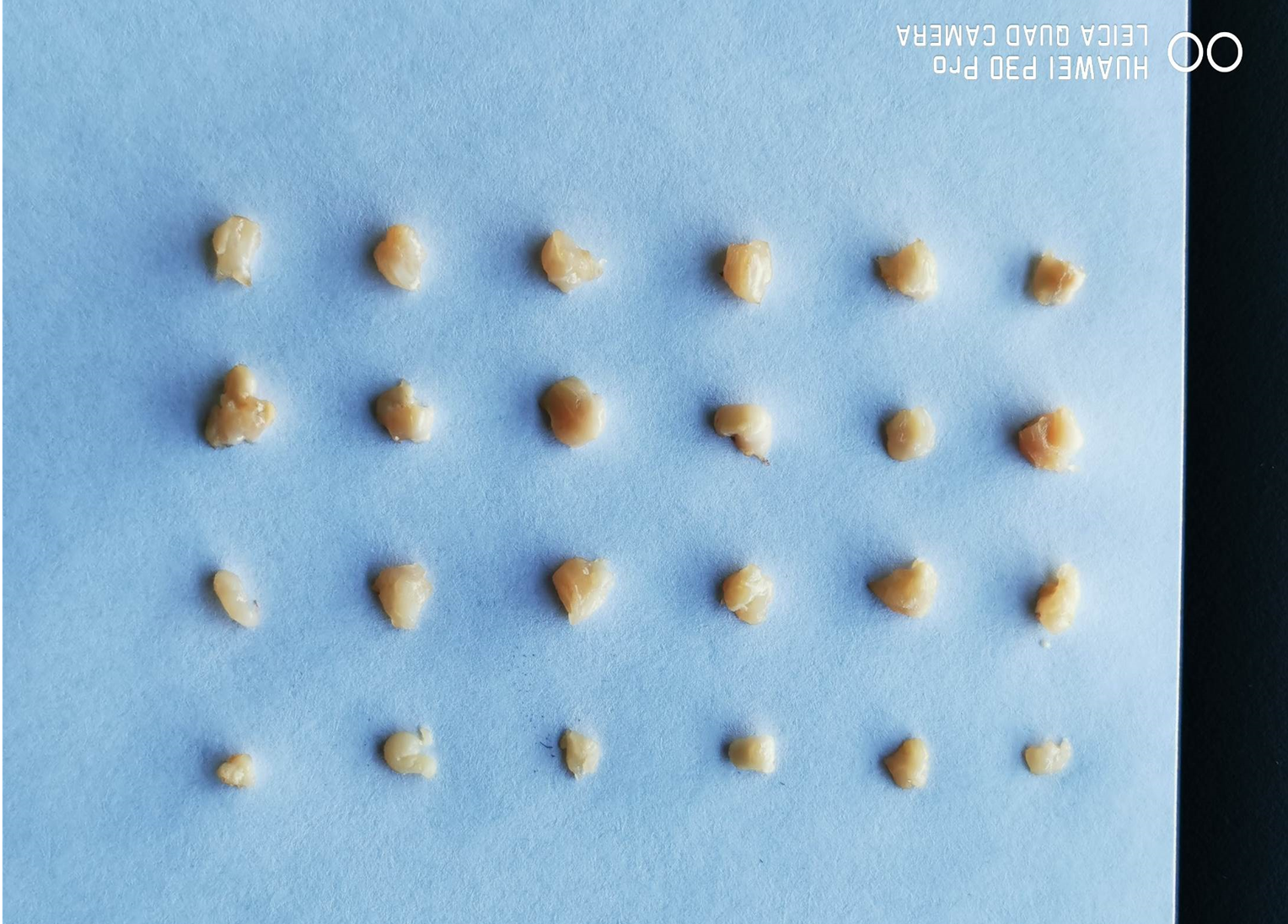

Supplement: Supplementary file 4 [file Image1.PNG]

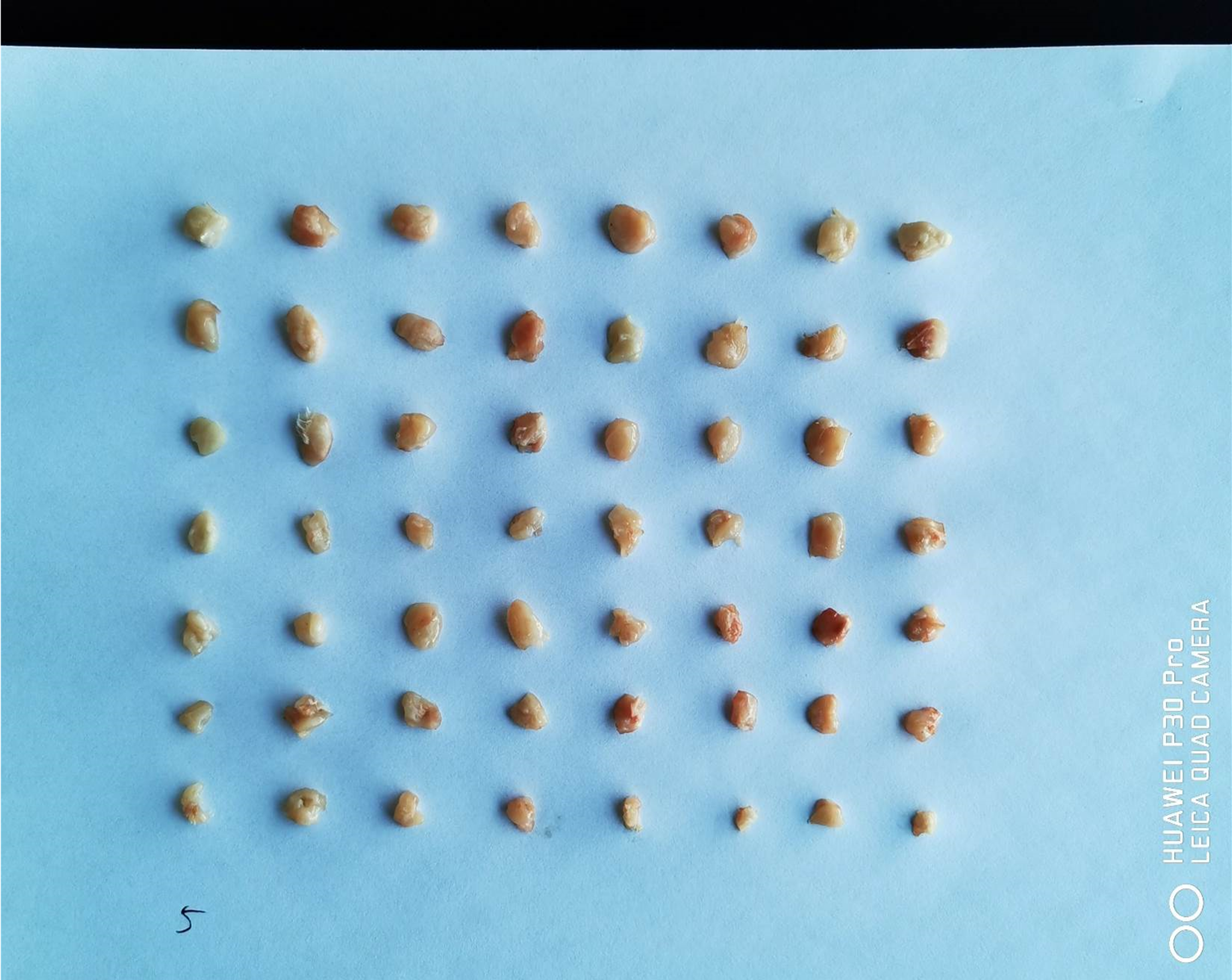

Supplement: Supplementary file 5 [file Image3.PNG]
